# NEP_1-40_-modified human serum albumin nanoparticles enhance the therapeutic effect of methylprednisolone against spinal cord injury

**DOI:** 10.1186/s12951-019-0449-3

**Published:** 2019-01-22

**Authors:** Yan Lin, Chunhong Li, Jian Li, Ruolan Deng, Juan Huang, Qinglian Zhang, Jiayao Lyu, Na Hao, Zhirong Zhong

**Affiliations:** 1 Department of Pharmaceutical Sciences, School of Pharmacy, Southwest Medical University, Luzhou, 646000 China; 2 Luzhou TCM Hospital, Luzhou, 646000 China; 3 Key Laboratory of Drug Targeting and Drug Delivery System, Ministry of Education (Sichuan University), Chengdu, 610000 China; 4 Key Laboratory of Medical Electrophysiology, Ministry of Education, Institute of Cardiovascular Research of Southwest Medical University, Luzhou, 646000 China

**Keywords:** NEP_1-40_, Human serum albumin nanoparticles, Methylprednisolone, Spinal cord injury

## Abstract

**Background:**

Frequent injection of high-dose methylprednisolone (MP) is used to treat spinal cord injury (SCI), but free MP is associated with various side effects and its water solubility is low, limiting potential dosing regimes and administration routes. Albumin-based nanoparticles, which can encapsulate therapeutic drugs and release cargo in a controlled pattern, show high biocompatibility and low toxicity. The Nogo protein, expressed on the surface of oligodendrocytes, can inhibit axonal growth by binding with the axonal Nogo receptor (NgR). Peptide NEP_1-40_, an NgR antagonist, can bind specifically to Nogo, significantly improving functional recovery and axon growth in the corticospinal tract. Therefore, we hypothesized that delivering MP within nanoparticles decorated with NEP_1-40_ could avoid the disadvantages of free MP and enhance its therapeutic efficacy against SCI.

**Results:**

We used human serum albumin to prepare MP-loaded NPs (MP-NPs), to whose surface we conjugated NEP_1-40_ to form NEP_1-40_-MP-NPs. Transmission electron microscopy indicated successful formation of nanoparticles. NEP_1-40_-MP-NPs were taken up significantly better than MP-NPs by the Nogo-positive cell line RSC-96 and were associated with significantly higher Basso–Beattie–Bresnahan locomotor scores in rats recovering from SCI. Micro-computed tomography assay showed that NEP_1-40_-MP-NPs mitigated SCI-associated loss of bone mineral density and accelerated spinal cord repair.

**Conclusions:**

NEP_1-40_-MP-NPs can enhance the therapeutic effects of MP against SCI. This novel platform may also be useful for delivering other types of drugs. 
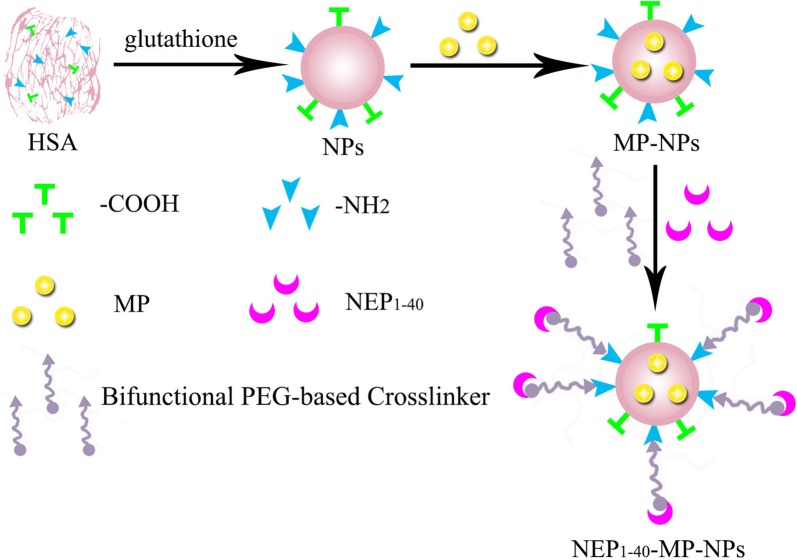

**Electronic supplementary material:**

The online version of this article (10.1186/s12951-019-0449-3) contains supplementary material, which is available to authorized users.

## Background

Spinal cord injury (SCI) often leads to impairment of motor, sensory, and autonomous functions [1], as well as neuronal loss, axonal degeneration, severe neurological deficits, and life-long movement and sensory dysfunction [2, 3]. Statistics from the US indicate that in 2016, approximately 282,000 people were affected by SCI, caused mainly by traffic accidents or falling from heights [4]. SCI has no effective treatment, reflecting its complex and poorly understood mechanisms [5]. Therefore, effective strategies are urgently needed to treat SCI.

High-dose methylprednisolone (MP) has shown neuroprotective efficacy in multicenter trials for limiting injury in SCI [6–8]. MP acts as an antioxidant and anti-inflammatory agent that can also stabilize lysosomal membranes and suppress edema [9–11]. MP can also inhibit apoptosis in oligodendrocytes [12, 13]. On the other hand, high-dose MP can trigger several side effects [14], such as pneumonia [15], gastric ulcer, leukemia, infection and neuropathy [16]. In addition, frequent intravenous injections affect the patient’s quality of life. MP shows poor water solubility, which limits the potential dosing regimes and administration routes that can be used.

It may be possible to bypass the disadvantages of free MP by encapsulating it within nanoparticles (NPs) that target the central nervous system (CNS). Indeed, various nanoparticle formulations have been assessed for SCI therapy [14, 17, 18], and some may seal damaged axonal membranes or activate intrinsic anti-oxidation pathways [19–21]. One promising endogenous nanoparticle is human serum albumin (HSA) [22], which is non-toxic, non-antigenic, biocompatible, and biodegradable, and it can bind drugs [23]. Given the abundance and robustness of HSA-NPs [24], many researchers have been trying to load them with therapeutic drugs. HSA-NPs are phagocytosed mainly by macrophages in the reticuloendothelial system, liver, kidney and bone marrow, allowing them to target organs [25]. The abundant free amino acids on the surface of HSA-NPs can be modified with ligands to increase targeting abilities [26, 27].

A promising candidate for targeting NPs to the CNS is the NEP_1-40_ peptide, which competes with the ability of Nogo, expressed on the surface of oligodendrocytes, to bind to the Nogo receptor (NgR) on axons [28, 29]. Nogo inhibits myelin-derived axon outgrowth and may hinder axonal regeneration following CNS injury [28]. NEP_1-40_ blocks Nogo-mediated inhibition of axonal outgrowth in the corticospinal tract, improving functional recovery, making it a potential treatment in SCI [28, 30, 31].

We hypothesized that we could enhance the therapeutic efficacy of MP and reduce its toxic effects by encapsulating it within NPs decorated with NEP_1-40_. We reasoned that this peptide should result in more specific NP targeting than other ligands [30, 31], and the peptide itself has been shown to promote neuronal regeneration in SCI. As a carrier for preparing MP-loaded NPs, we chose human serum albumin, which may be more biocompatible and less toxic than other carriers.

## Results

### Characterization of NPs preparations

**Fig. 1:**
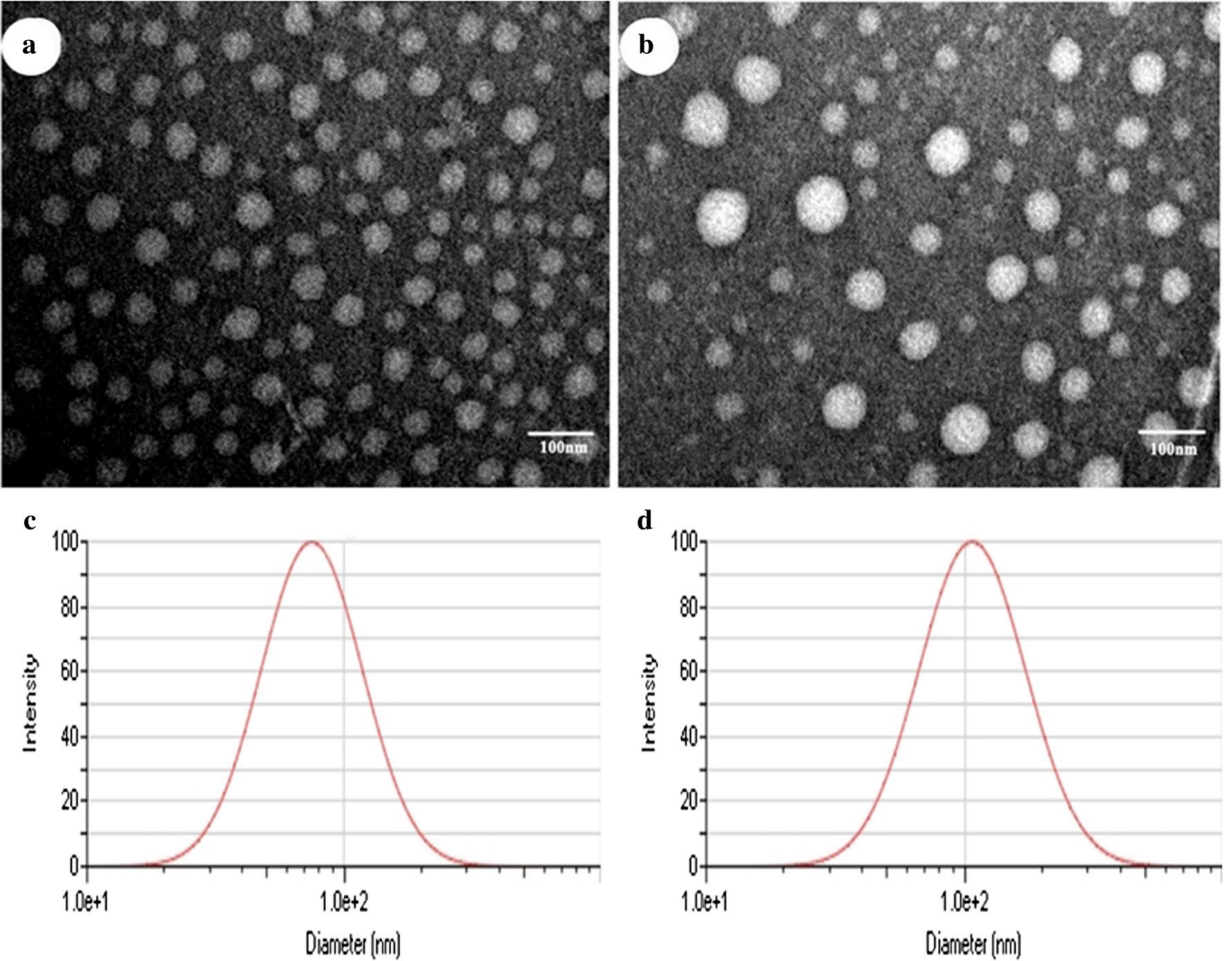
Morphology of blank NPs (**a**) and MP-NPs (**b**) observed by transmission electron microscopy. Size distribution of blank NPs (**c**) and MP-NPs (**d**) detected by dynamic light scattering

Blank NPs, MP-loaded NPs (MP-NPs) and NEP_1-40_-modified MP-NPs (NEP1-40-MP-NPs) were prepared as described in Methods and then characterized using a wide variety of techniques. Blank NPs showed a size of 74.87 ± 0.19 nm and polydispersity index (PDI) of 0.225 ± 0.003 (Table 1). MP loading significantly increased NP size (P < 0.001): MP-NPs showed a size of 107.76 ± 1.68 nm and NEP1-40-MP-NPs, a size of 192.19 ± 4.43 nm. NEP1-40-MP-NPs were significantly larger than MP-NPs (P < 0.001). Moreover, all types of NPs showed PDIs < 0.3, suggesting homogeneity. Transmission electron microscopy showed that NPs were spherical, MP-NPs were larger than blank NPs, and both NP preparations showed a uniform size distribution (Fig. 1).

**Table 1:** Size distribution and zeta potential of nanoparticles

	Particle size (nm)	Zeta potential (mV)	PDI
Blank NPs	74.87 ± 0.19	21.26 ± 0.73	0.225 ± 0.003
MP-NPs	107.76 ± 1.68*	20.53 ± 1.34	0.251 ± 0.001
NEP_1-40_-MP-NPs	192.19 ± 4.43^#^	− 8.04 ± 3.99	0.263 ± 0.002

Results are expressed as mean ± SD from three independent experiments

*MP* methylprednisolone, *NP* nanoparticle, *PDI* polydispersity index

* P < 0.0001 vs. MP-NPs and ^#^ P < 0.0001 vs. NEP_1-40_-MP-NPs

**Fig. 2:**
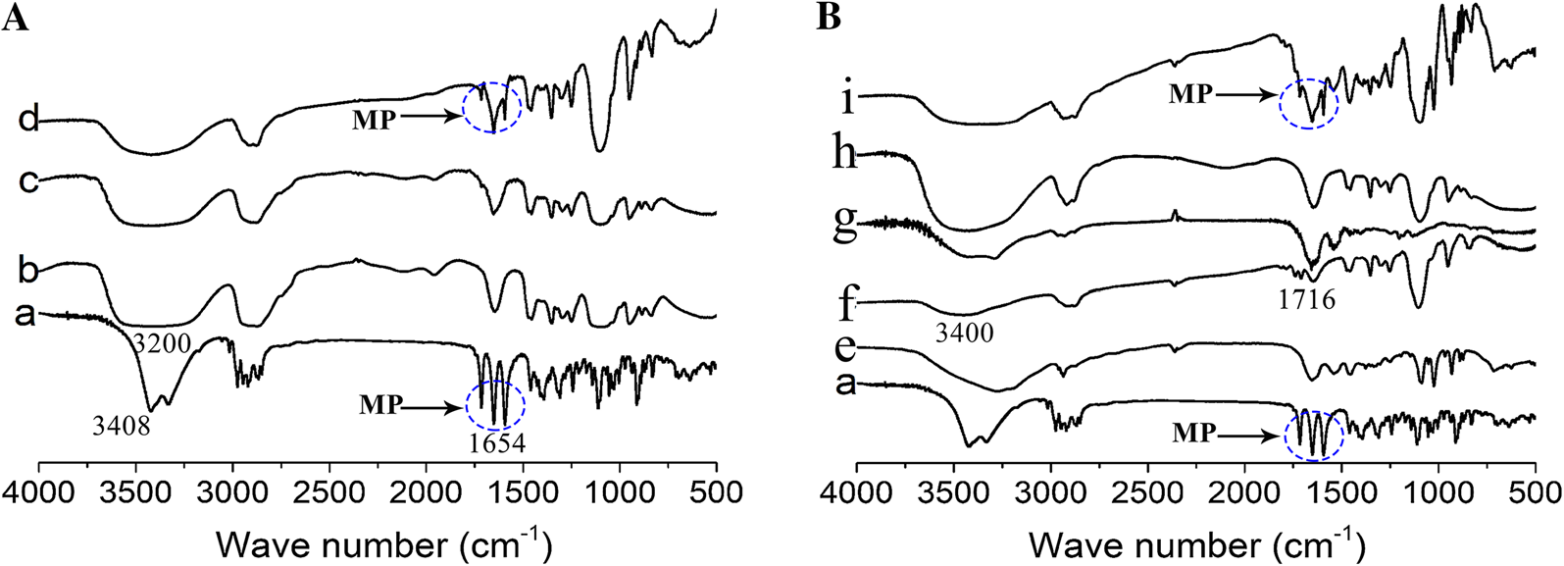
Fourier-transform infrared spectra of NPs. **A** Spectra of MP (trace *a*), PEG (trace *b*), MP-PEG (trace *c*), and the physical mixture of MP and PEG (trace *d*). Peaks at 3408, 1654 and 3200 cm^−1^ corresponded, respectively, to the free υ_O–H_, conjugated υ_C=O_ and connective υ_O–H_. **B** Spectra of MP (trace *a*), blank NPs (trace *e*), NHS-PEG5000-MAL (trace *f*), NEP_1-40_ (trace *g*), NEP1-40-MP-NPs (trace *h*), and the physical mixture of MP, NHS-PEG5000-MAL and NEP_1-40_ (trace *i*). Peaks at 1716 and 3400 cm^−1^ corresponded, respectively, to amino ester υ_C = O_ and connective υ_O–H_

Fourier-transform infrared spectroscopy confirmed the formation of MP-NPs (Fig. 2A) and NEP_1-40_-MP-NPs (Fig. 2B). A peak due to vibrations of the conjugated C=O bond in MP was detected around 1654 cm^−1^ in the spectra of MP, the physical mixture of MP and PEG, and the physical mixture of MP, NHS-PEG5000-MAL and NEP_1-40_ (Fig. 2A, B, traces a, d, i). Additional peaks were seen in the region of 3250–3408 cm^−1^ corresponding to the O–H bond in MP (Fig. 2A, trace a) and around 3200 cm^−1^ corresponding to the vibration of the O–H bond in PEG (Fig. 2A, trace b). The MP peak around 1654 cm^−1^ was not found in the spectrum of MP-PEG (Fig. 2A, trace c), which was almost identical to that of PEG (Fig. 2A, trace b). The physical mixture of MP and PEG contained characteristic features of the spectra of PEG and MP on their own (Fig. 2A, trace d), suggesting that MP was encapsulated inside PEG to form MP-PEG.

We did not observe a peak for vibration of the unsaturated bond (υ_C-H_) at 3000-3100 cm^−1^ in the spectrum of NHS-PEG_5000_-MAL (Fig. 2B, trace *f*). This may be because the peak was obscured by the υ_O–H_ peak around 3400 cm^−1^. We observed a peak at 1716 cm^−1^ corresponding to υ_C=O_ of the amino ester in NHS-PEG_5000_-MAL (Fig. 2B, trace *f*), which was absent from the spectrum of NEPI-40-MP-NPs (Fig. 2B, trace *h*). The MP peak around 1654 cm^−1^ was present in the spectrum of the physical mixture of MP, NHS-PEG5000-MAL and NEP_1-40_ (Fig. 2B, trace *i*), but it was absent from the spectrum of NEPI-40-MP-NPs (Fig. 2B, trace *h*). These results suggest that MP was successfully wrapped in nanoparticles, and that MP-NPs successfully reacted with NHS-PEG_5000_-MAL.

Infrared spectroscopy could not be used to analyze NEP_1-40_ or MP-NPs on their own. Nevertheless, HPLC and laser confocal microscopy studies confirmed that the NEP_1-40_-MP-NP delivery system was successfully established. The conjugation of NEP_1-40_ with MP-NPs to form NEP_1-40_-MP-NPs was confirmed by fluorescently labeling the peptide and then measuring the fluorescence signal of sample retained by the filter and of flow-through that passed through the filter (Additional file 1: Figure S1). The retained sample was re-filtered a total of five times. Fluorescence intensity (arbitrary units) of the sample decreased from 2938.67 ± 61.85 after the first filtration to 647.47 ± 2.52 after the fifth. The fluorescence intensity of the flow-through was 1397.33 ± 188.54 after the first filtration and became undetectable after the fifth filtration. These results suggest that NEP_1-40_ was successfully conjugated with MP-NPs to form NEP_1-40_-MP-NPs. The conjugation rate was calculated to be 70 ± 1.3% (see “Methods”).

As a complementary method to fluorescence detection, we quantified the conjugation rate using HPLC. Under our conditions, NEP_1-40_ eluted as a sharp peak with a retention time of 21 min. In contrast, no such peak was detected in the filtrate from the fifth ultrafiltration. The conjugation rate, 71 ± 0.8%, agreed well with that determined based on fluorescence detection.

### Drug encapsulation, loading rate and release in vitro by NPs

**Fig. 3:**
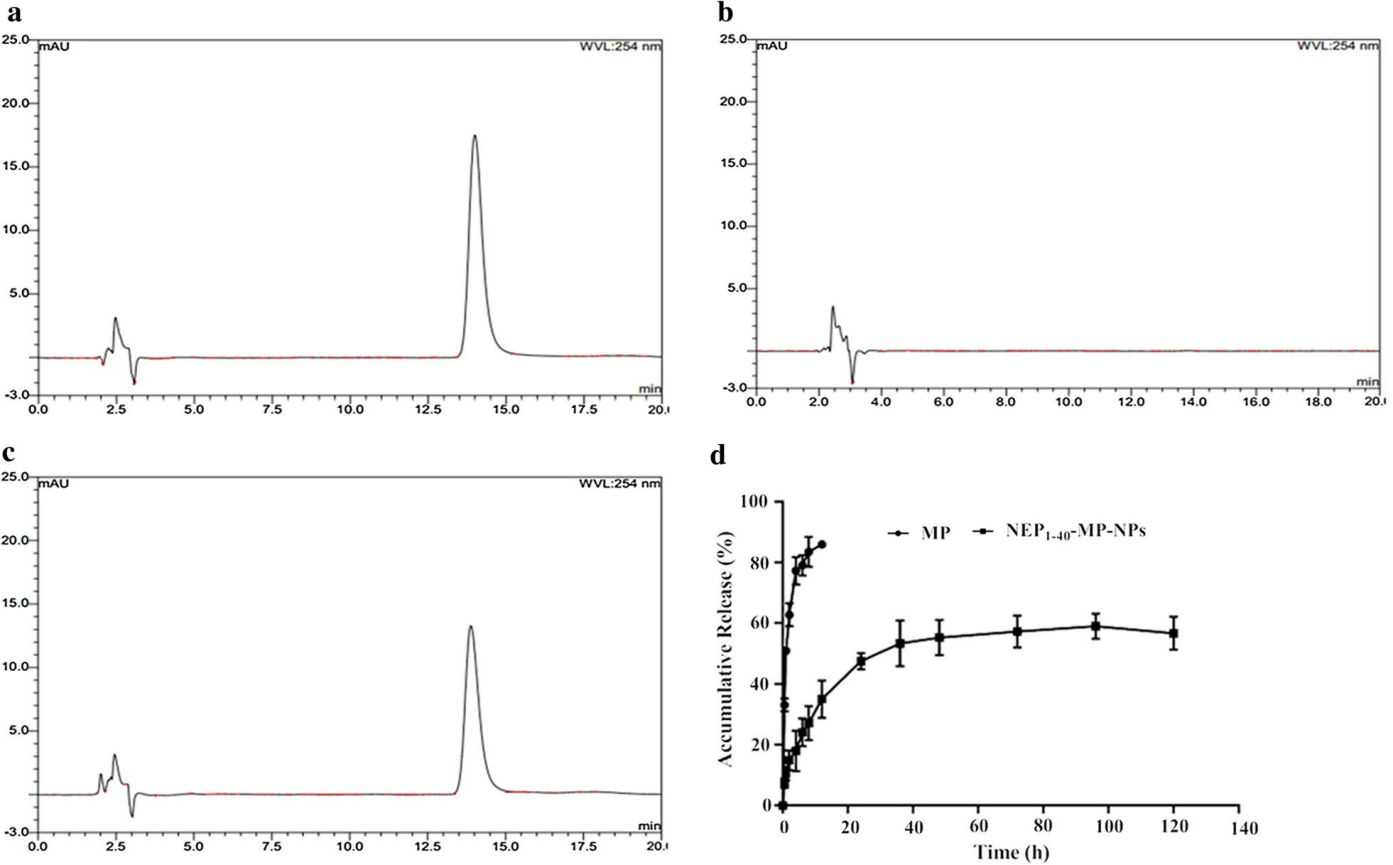
Cumulative release of MP from NEP_1-40_-MP-NPs and free MP determined by HPLC. Representative chromatograms of **a** MP, **b** blank NPs, **c** MP-NPs. **d** Quantitation of cumulative release (mean ± SD, n = 3)

MP-NPs showed an encapsulation efficiency of 84.06% ± 3.48% and drug-loading rate of 7.32% ± 0.71%. Cumulative release in vitro during the first 12 h was 59.05 ± 4.08% for NEP_1-40_-MP-NPs and ~ 85% for free MP (Fig. 3). NEP_1-40_-MP-NPs showed sustained release without a significant early burst, suggesting that MP was completely encapsulated within nanoparticles rather than being adsorbed on the surface of nanoparticles.

We examined various kinetics models to describe NEP_1-40_-MP-NPs release in vitro. The models gave the following results: zero-order equation, Q = 0.4097 t + 22.3559 (R^2^ = 0.6551); first-level equation, Q = 56.6237 (1−e^−0.0887 t^) (R^2^ = 0.9739); Higuchi equation, Q = 5.3239 t^1/2^ + 10.9399 (R^2^ = 0.8609); and Ritger–Peppas equation, Q = 12.7675 t^0.3525^ (R^2^ = 0.9413). We concluded that the first-level equation best described NEP_1-40_-MP-NPs release in PBS (pH 7.4) in vitro.

### In vitro toxicity detection by hemolysis assay

The hemolytic assay is regarded as a sensitive method to evaluate NPs biosafety. In our study, we sought to evaluate the hemolytic activity of NEP_1-40_-MP-NPs. In the positive control, ~ 100% hemolysis was observed, compared to only 4.85% for NEP_1-40_-MP-NPs at concentrations up to 2 mg/mL (Additional file 1: Figure S2). This low level is within the normal range recommended by the ASTM E2524-08 standard. These results demonstrate that NEP_1-40_-MP-NPs have good blood biocompatibility and may be safe for clinical application.

### Cellular uptake of NEP_1-40_-MP-NPs

**Fig. 4:**
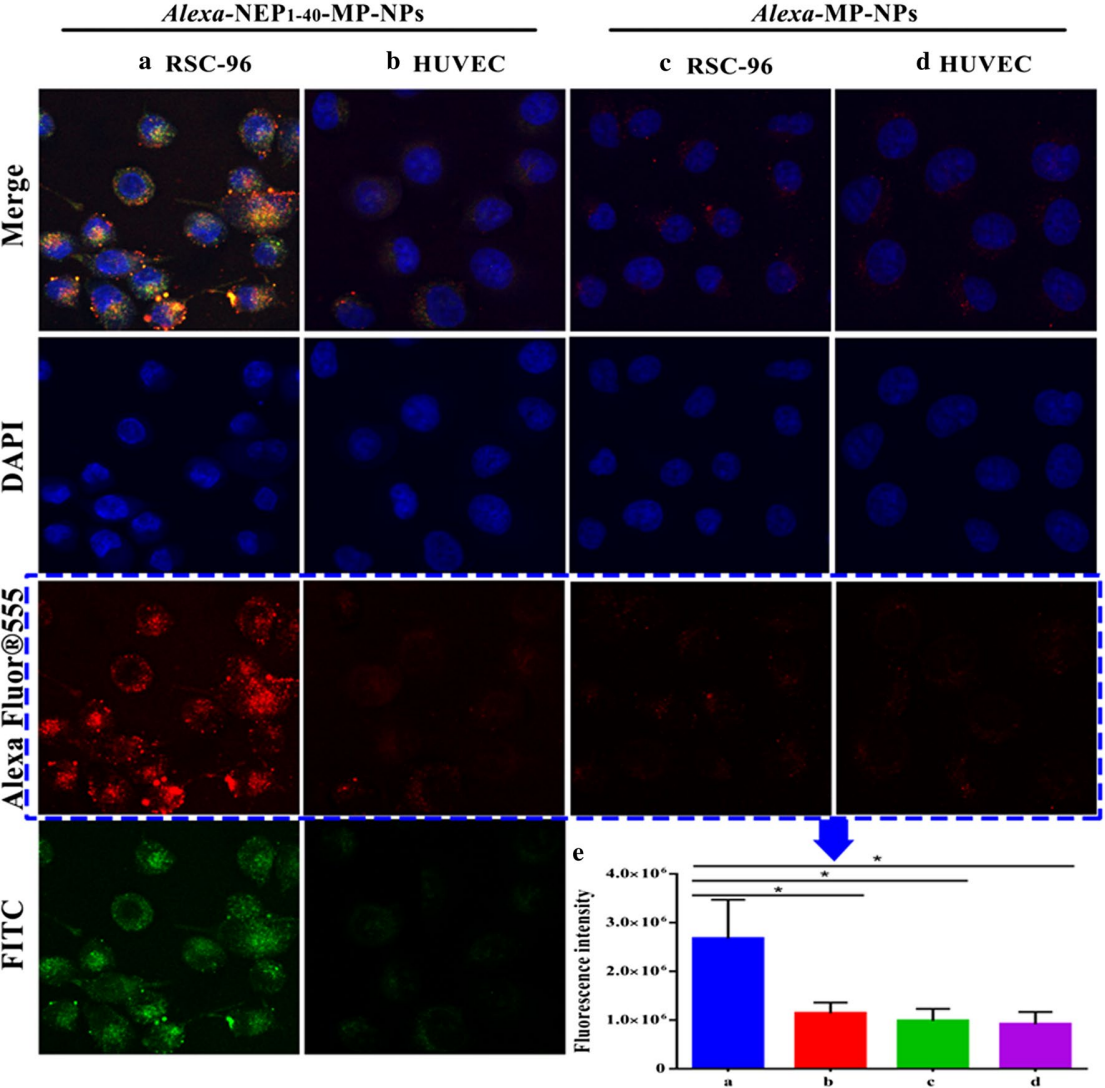
Cellular uptake of NPs by RSC-96 cells and HUVECs. Fluorescently labeled NEP_1-40_ appeared green; fluorescently labeled NPs, red. Representative images showing **a** RSC-96 cells or **b** HUVECs treated with NEP_1-40_-MP-NPs. Representative images showing **c** RSC-96 cells or **d** HUVECs treated with MP-NPs. **e** Summary of red fluorescence intensity analyzed by NIH Image-Pro Plus 6.0. Results are mean ± SD (n = 3)

Uptake of free NEP_1-40_ and NEP_1-40_-MP-NPs by Nogo-positive RSC-96 cells was analyzed. As a negative control, we performed the same experiments in Nogo-negative human umbilical vein endothelial cells (HUVECs). In these studies, NEP_1-40_ was labeled with FITC that could be detected in the green channel, while MP-NPs were labeled with Alexa Fluor^®^ 555 that could be detected in the green channel. Co-localized red and green puncta were observed in cells treated with NEP_1-40_-MP-NPs, suggesting that the peptide had successfully conjugated with MP-NPs (Fig. 4a). These intracellular fluorescent signals were much stronger in RSC-96 cells than in HUVECs (Fig. 4b). No fluorescence signal was detected from RSC-96 or HUVECs treated with MP-NPs (Fig. 4c, d). These results were confirmed in quantitative analysis (Fig. 4e). These results suggest that NEP_1-40_ significantly enhances NP uptake specifically in Nogo-positive cells.

### Therapeutic efficacy of NEP_1-40_-MP-NPs against SCI in a rat model of SCI

**Fig. 5:**
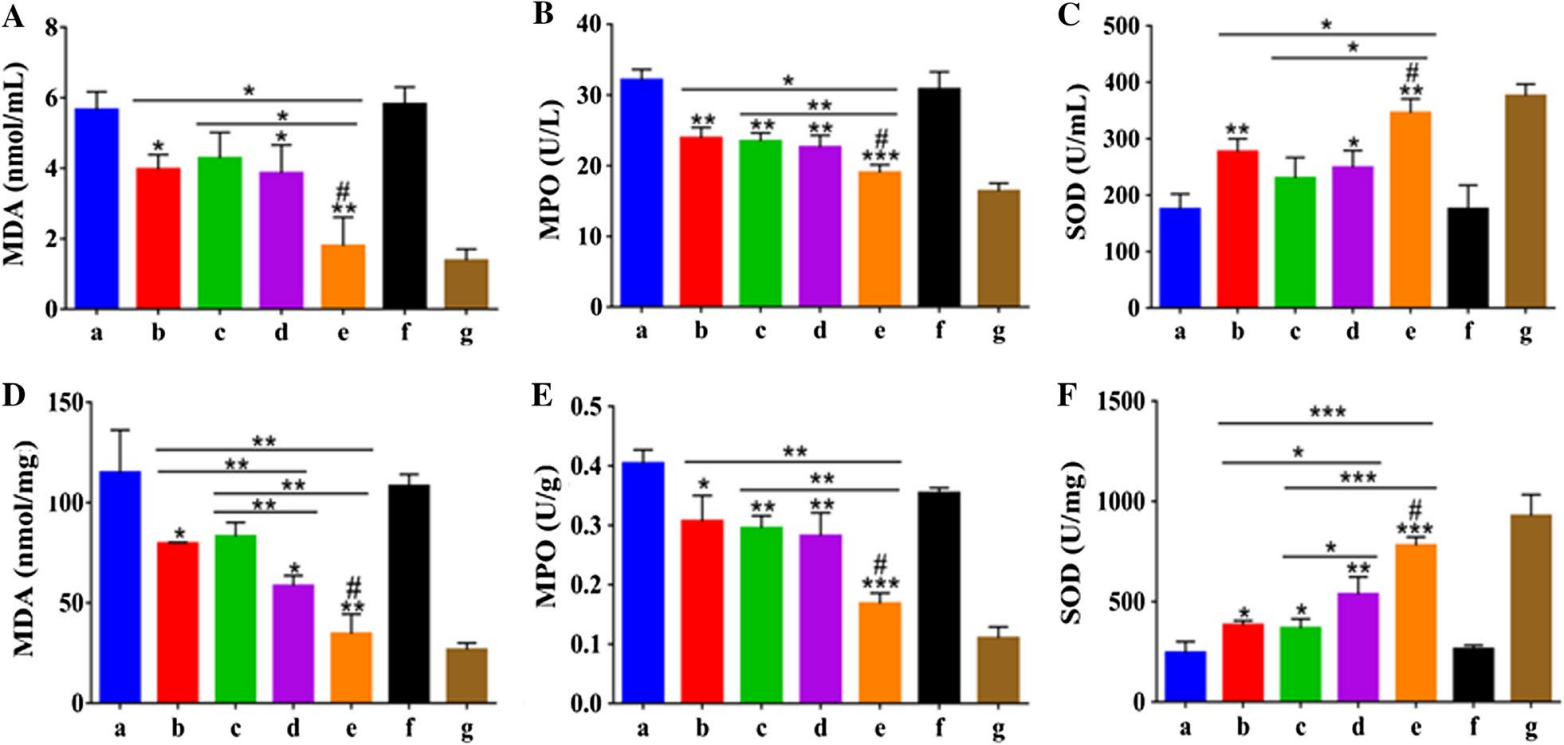
Malondialdehyde (MDA), myeloperoxidase (MPO) and superoxide dismutase (SOD) levels in serum and spinal cord tissue after SCI in rats. **A** MDA in serum. **B** MPO in serum. **C** SOD in serum. **D** MDA in spinal cord. **E** MPO in spinal cord. **F** SOD in spinal cord. Animals were treated with (a) saline, (b) control MPS, (c) MP, (d) MP-NPs, (e) NEP_1-40_-MP-NPs, (f) blank NPs, or (g) sham-operated. *P < 0.05, **P < 0.01, and ***P < 0.001 vs. saline; ^#^P < 0.05 vs. MP-NPs. Data are mean ± SD (n ≥ 3)

Levels of malondialdehyde (MDA) are an important indicator of lipid peroxidation by free radicals. MDA levels in serum and spinal cord were significantly lower in SCI rats treated with MPS, MP-NPs or NEP_1-40_-MP-NPs than in saline-treated controls (Fig. 5A, D), in which methylprednisolone sodium succinate (MPS) was taking as the positive control. Levels in the NEP_1-40_-MP-NP group were significantly lower than in the other treatment groups. These results indicate that MPS and MP encapsulated in NPs can alleviate free radical damage in rats after SCI, with NEP_1-40_-MP-NPs showing the greatest effect.

Levels of myeloperoxidase (MPO) are an indicator of polymorphonuclear leukocyte accumulation and therefore of inflammation severity. Levels of MPO were significantly lower in animals treated with NEP_1-40_-MP-NPs than in other treatment groups (Fig. 5B, E). These results indicate that free MP and MP encapsulated in NPs can alleviate the inflammatory response after SCI, with NEP_1-40_-MP-NPs showing the greatest effect.

Superoxide dismutase (SOD) is a key enzyme in cellular antioxidant systems. SOD levels in serum and spinal cord tissue were significantly higher in animals treated with free MP, MPS, MP-NPs or NEP_1-40_-MP-NPs than in saline-treated controls (Fig. 5C, F). Levels in the NEP_1-40_-MP-NP group were significantly higher than in the other treatment groups. These results suggested that MP, MPS, MP-NPs or NEP_1-40_-MP-NPs enhanced antioxidant capacity after spinal cord injury, with NEP_1-40_-MP-NPs showing the strongest improvement.

**Fig. 6:**
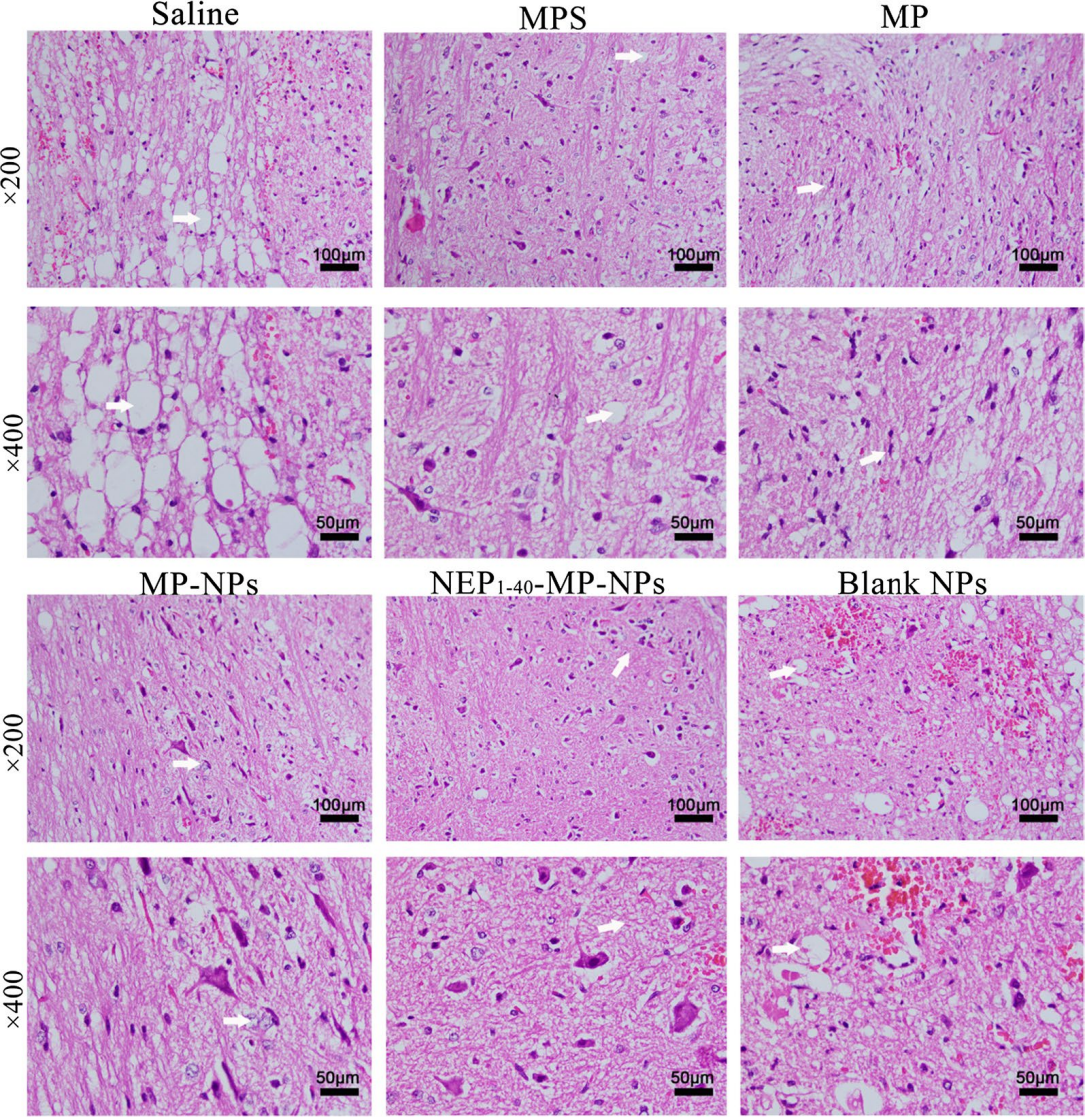
Representative images showing histopathological analysis in spinal cord tissue after spinal cord injury at 1 day after intrathecal injection of saline, MPS (control), MP, MP-NPs, NEP_1-40_-MP-NPs, or blank NPs (n = 3). Bold arrows indicate the location of lesions

Histopathology of spinal cord tissue at 1 day after the various treatments indicated that in animals treated with saline or blank NPs, neuronal nuclei and cell bodies were shrunken, synapses were reduced and ruptured, and hollow-like cell necrosis was observed (Fig. 6). Neurons were edematous and fewer in number, and they showed morphological changes. A large area of hemorrhage was observed in gray and white matter. Tissue from animals treated with MP, MPS, MP-NPs or NEP_1-40_-MP-NPs showed more numerous neuronal cells and smaller hemorrhage area, with NEP_1-40_-MP-NPs giving the best results.

**Fig. 7:**
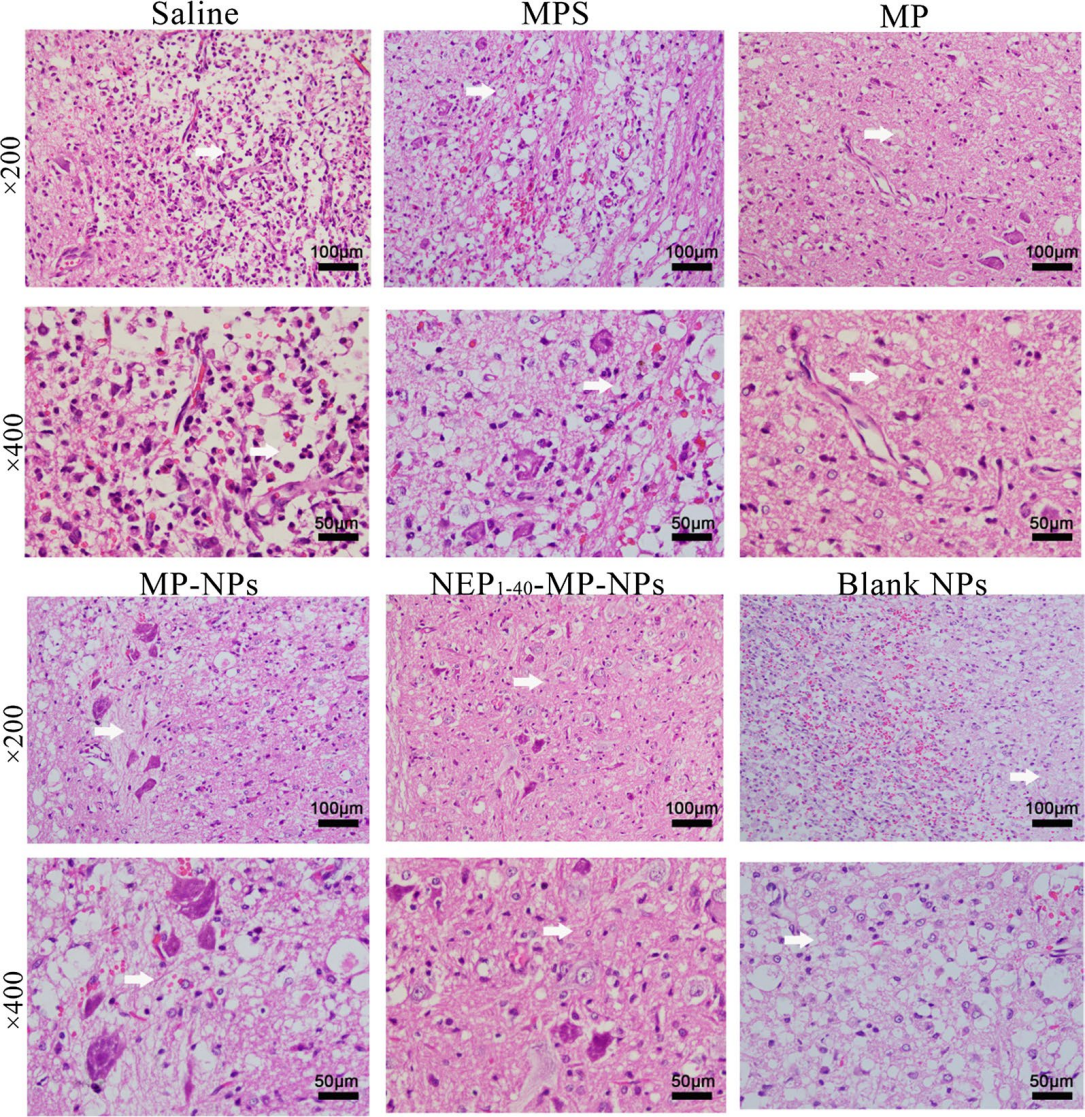
Representative images showing histopathological analysis in spinal cord tissue after spinal cord injury at 3 days after intrathecal injection of saline, MPS (control), MP, MP-NPs, NEP_1-40_-MP-NPs, or blank NPs (n = 3). Bold arrows indicate the location of lesions

Histopathology at 3 days after the various treatments showed necrosis of neuronal cells, exudation of inflammatory cells and proliferation of macrophages in the central area of the injury and in adjacent gray and white matter in animals treated with saline or blank NPs (Fig. 7). The number of inflammatory cells was reduced in animals treated with NEP_1-40_-MP-NPs, MP, MPS or MP-NPs. NEP_1-40_-MP-NP treatment was associated with the smallest number of inflammatory cells and greatest numbers of neuronal and glial cells.

**Fig. 8:**
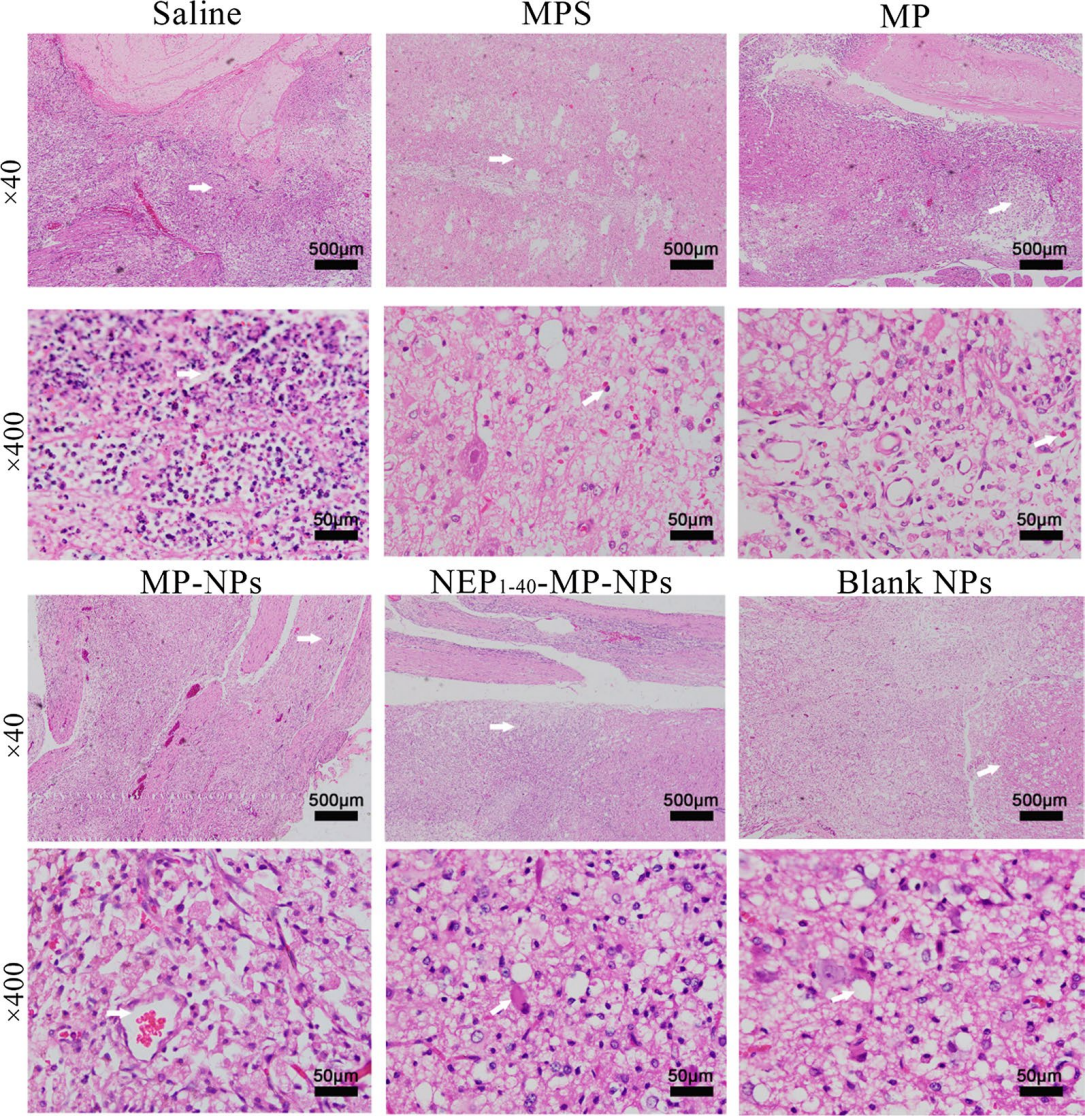
Representative images showing histopathological analysis in spinal cord tissue after spinal cord injury at 7 days after intrathecal injection of saline, MPS (control), MP, MP-NPs, NEP_1-40_-MP-NPs, or blank NPs (n = 3). Bold arrows indicate the location of lesions

Histopathology at 7 days after the various treatments showed vessel reconstruction in the central area of the lesion and adjacent sites, with scattered hemorrhage areas in animals treated with saline or blank NPs (Fig. 8). Hyperplasia of macrophages, necrosis of peripheral nerve cells, appearance of autophagosomes and foam cells were also observed. Treatment with NEP_1-40_-MP-NPs, MP, MPS or MP-NPs showed improved proliferation of astrocytes and fibroblasts as well as reduced numbers of foam cells. Tissue from animals treated with NEP_1-40_-MP-NPs group showed the greatest proliferation of glial cells and glial junction formation as well as smallest number of autophagosomes and least extensive scar formation. These results suggest that NEP_1-40_-MP-NPs were the most effective at shortening the repair period after SCI.

**Fig. 9:**
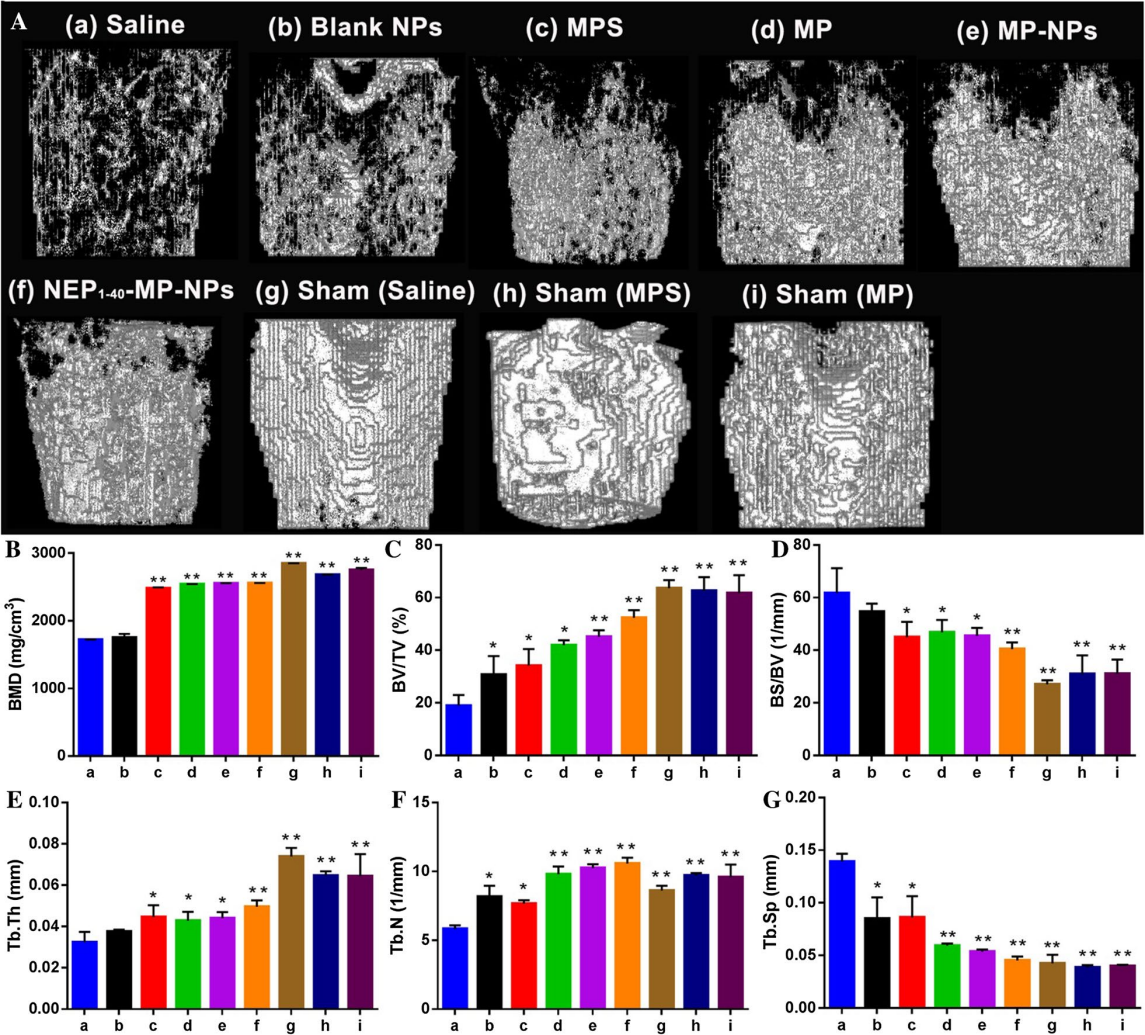
Effects of the different formulations on trabecular bone microarchitecture in rats after spinal cord injury. **A** Representative micro-computed tomographs after treatment with (a) saline, (b) blank NPs, (c) control MPS, (d) MP, (e) MP-NPs, (f) NEP_1-40_-MP-NPs, (g) saline sham, (h) MPS sham and (i) MP sham. **B** Bone mineral density (BMD). **C** Bone volume/total volume (BV/TV). **D** Bone surface area/bone volume (BS/BV). **E** Trabecular thickness (Tb.Th). **F** Trabecular number (Tb.N). **G** Trabecular spacing (Tb.Sp). *P < 0.05 or **P < 0.01 vs. saline. Data are mean ± SD (n = 3)

Next we analyzed effects of the various treatments on trabecular bone microstructure using micro-computed tomography because SCI can induce osteoporosis [45, 46]. All treatment groups showed lower bone mineral density and bone thickness than in the sham group (Fig. 9A). Meanwhile, compared with the sham group, all the other treatment groups showed decreased number of bone trabeculae and the bones showed fractures and disruptions in the marrow cavity. Several bone microstructure parameters were significantly different between saline- and sham-treated animals as a result of SCI (Fig. 9B–G). While all parameters were similar between MP and MPS groups, many parameters differed between these groups and the NEP_1-40_-MP-NP group. These results suggest that NEP_1-40_-MP-NPs can promote restoration of bone microarchitecture after SCI.

**Fig. 10:**
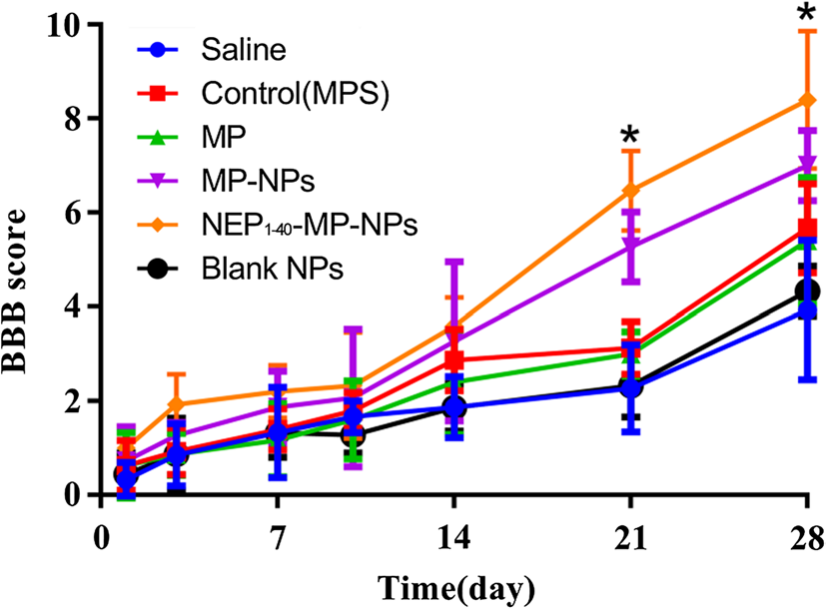
Hind limb neuromotor function in rats after spinal cord injury as determined by the Basso–Beattie–Bresnahan locomotor score. Data are mean ± SD (n = 3). *P < 0.05 vs. MP-NPs

Finally, we assessed the effects of the various treatments on hind limb neuromotor function in SCI rats (Fig. 10). Basso-Beattie-Bresnahan locomotor scores peaked on day 21 in the sham group (data not shown). At 1 day after SCI, scores were similar among all groups, but from day 14 onwards, scores increased gradually in the NEP_1-40_-MP-NP and MP-NP groups. Scores on day 28 were 8 ± 1.461 in rats treated with NEP_1-40_-MP-NPs and 7 ± 0.745 in animals treated with MP-NPs, significantly better than in other treatment groups (P < 0.05). Scores were significantly higher in the NEP_1-40_-MP-NP group than in the MP-NP group.

## Discussion

In the present study, we developed a novel NEP_1-40_-MP-NP drug delivery system for enhancing the therapeutic effects of MP against SCI while allowing the use of lower doses, leading to lower side effects. Our MP-NPs were uniform and spherical and showed high encapsulation efficiency. Release of MP in vitro from NEP_1-40_-MP-NPs was sustained and significantly slower than the release of free MP. These results agree with a previous report that NPs can serve as a drug reservoir, releasing the compounds in sustained manner [23–25]. NEP_1-40_-MP-NPs were more effective than MP-NPs at reducing secondary spinal cord damage and loss of bone mineral density in a rat model of SCI, and the decorated NPs accelerated the repair process. We speculate that the ability of NEP_1-40_-MP-NPs to enhance treatment effects of MP against SCI is because of the drug’s slow release and NEP_1-40_-mediated targeting of oligodendrocytes.

MP-NPs were prepared using blank HSA nanoparticles as the substructure of the core matrix. Critical to proper NP assembly was the manner in which materials were added, as well as the ratio of ethanol to water when disulfide bonds were formed in stabilized HSA-NPs. Glutathione was used as an effective reducing agent to break intra-molecular disulfide bonds in HSA, and it was maintained in the acid form by keeping pH low [26] through addition of HCl to the HSA solution and through dropwise addition to ethanol to reform inter-molecular disulfide bonds. These steps led to the formation of blank NPs, whose diameter was strongly affected by the ionic strength of the solution [33]. These blank NPs were exposed to air for at least 3 h with continuous stirring to enhance intermolecular disulfide bonds and glutathione oxidation; otherwise, nanoparticles quickly swelled up after adding water. These blank HSA-NPs were lyophilized in the presence of mannitol as a lyoprotectant. To this powder was added an MP-PEG compound that was prepared by mixing PEG_400_ and MP in ethanol, and then removing the ethanol. We hypothesize that MP-PEG formed through hydrogen bond formation as ethanol evaporated, since PEG_400_ has abundant hydrogen bond donors and acceptors. MP-NPs were prepared by adding MP-PEG liquid compound drop-wise to lyophilized blank HSA nanoparticles powder with stirring. The mixture was dispersed into acidified water. Due to the strong water polarity, hydrogen bonds between PEG and MP were broken, and the PEG formed new hydrogen bonds with the water. Because of the fluidity of MP-PEG and the affinity of HSA-NPs for hydrophobic drugs, MP readily entered the matrix structure of HSA-NPs. Since particle size is the main factor for ensuring homogeneous distribution of colloidal drug carrier systems [33], we subjected the mixture to intermittent ultrasound in a cold-water bath, forming milky white MP-NP solution. This desolvation technique is relatively simple and may allow better control of NEP_1-40_-MP-NP size than emulsification or supercritical anti-solvent processes involving albumin coating.

Our assays with MDA and SOD suggest that NEP_1-40_-MP-NPs can help scavenge free radicals. This may be analogous to the way in which nanoparticles based on cerium oxide, carbon, manganese, or platinum can scavenge reactive oxygen species [34]. Further work should assess the potential mechanism(s) of this activity by NEP_1-40_-MP-NPs in greater detail.

NEP_1-40_-MP-NPs showed good blood biocompatibility (Additional file 1: Figure S2) and therefore may be safe for clinical application, based on the ASTM E2524-08 standard. Nevertheless, MP-NPs and NEP_1-40_-MP-NPs showed some hemolytic effects. We speculate that this risk increases with higher NP concentration, since the probability of contacts between NPs and red blood cells increases.

This NEP_1-40_-MP-NP drug delivery system may provide a new strategy for using MP against SCI. It may also provide a new platform for delivering other insoluble drugs to the CNS and other tissues, depending on the specificity coating applied.

## Methods

### Materials

NEP_1-40_ was synthesized by Top-peptide (Shanghai, China). NHS-PEG_5000_-MAL was from Xi’an RuiXi (Xi’an, China). Ethanol, Tween-80 and PEG_400_ were obtained from Chengdu Kelon Chemical Reagent Factory (Chengdu, China). Human serum albumin, l-glutathione (98%), MP and 4′,6-diamidino-2-phenylindole (DAPI) were provided by Solarbio (Beijing, China). Dulbecco’s modified Eagle’s medium (DMEM) was obtained from Hammer Flew (Beijing, China). Fetal bovine serum was purchased from TianHang (Huzhou, China). Trypsin and Alexa Fluor^®^ 555 were provided by Thermo Fisher Scientific (Waltham, MA, USA). Paraformaldehyde was obtained from Jinshan Chemical Company (Chengdu, China). MPO, SOD and MDA kits were purchased from the Nanjing Jiancheng Bioengineering Institute (Jiangshu, China).

### Cells and animals

Rat Schwann cell line RSC-96 [35] and HUVECs were cultured in DMEM with fetal bovine serum (10%, v/v), penicillin (100 U/mL), and streptomycin (100 μg/mL). Cells were cultured at 37 °C in 5% CO_2_.

Eight- to ten-week-old female Sprague–Dawley rats (150–220 g) were purchased from the Laboratory Animal Center of Southwest Medical University (Luzhou, China) and kept in controlled humidity, temperature, and lighting conditions. All animal experiments were approved by the Animal Ethics Committee of Southwest Medical University (permit 2016101101), and all animal operations were performed according to the guidelines of the Local Animal Use and Care Committee of Luzhou (Sichuan, China) and in accordance with the National Animal Welfare Law of China.

### Preparation of NEP_1-40_-MP-NPs

Blank NPs were prepared by desolvation [36, 37]. Briefly, glutathione powder (12 mg) was dissolved and mixed with 6 mL of HSA solution (5 mg/mL). The mixture was stirred slowly for 2 h at room temperature and adjusted to pH 3.45 using HCl solution (0.1 mol/L). The protein solution was then added to ethanol at 1 mL/min to form new intermolecular HSA disulfide bonds until the ethanol/water ratio was 3.5:1 (v/v). Then the solution was exposed to oxygen with stirring for at least 3 h to stabilize the newly formed disulfide bonds [32]. Finally, the suspension was diluted with 10 mL of distilled water, and the ethanol was removed under vacuum in a rotary evaporator at 37 °C. Excess glutathione was removed from the resulting suspension of blank nanoparticles by dialysis (mol. wt cut-off, 3500 Da) for 3 times (8 h each time). The suspension was then lyophilized with mannitol (5%) as lyoprotectant to obtain a powder of blank NPs.

To prepare the MP-PEG complex, 6 mg of MP and 100 μL of PEG_400_ were added into 4 mL of ethanol. Ethanol was removed under vacuum through rotary evaporation at 45 °C. MP-loaded NPs (MP-NPs) were prepared by ultrasonication. Briefly, 15 μL of the preformed MP-PEG complex was mixed with 15 mg of the blank NP powder and mixed with a small spatula. The mixture was dispersed into 5 mL of ultra-pure water. The dispersion was ultrasonicated intermittently at 90 W for 5 min using a probe ultrasonicator in a cold-water bath.

We prepared the peptide NEP_1-40_-modified MP-NPs (NEP_1-40_-MP-NPs) through sulfhydryl groups of NPs [35–38]. Briefly, to activate MP-NPs, the heterobifunctional cross linker of NHS-PEG_5000_-MAL (16 mg) was dissolved in 1 mL of phosphate buffer (pH 8.0) and mixed with nanoparticle suspension (100 mg) at a molar ratio of 2:1 based on previous work [39]. The mixture was incubated with shaking (550 rpm) for 1 h at room temperature. Then the activated MP-NPs were purified by two cycles of ultra-centrifugation at 15,000*g* for 8 min to remove un-reacted NHS-PEG_5000_-MAL. The pellet was then re-dispersed in 1 mL of phosphate buffer (pH 8.0) and incubated with NEP_1-40_ peptide (870 μg) at a molar ratio of 1:1 for 1 h with stirring (600 rpm); this molar ratio was based on a previous report that the terminal cysteine (Cys) of NEP_1-40_ reacts with maleimide in a molar ratio of 1:1 [39]. To remove excess small molecules, the reaction solution was centrifuged for 10 min at 6000*g* through an ultrafiltration centrifuge tube with molecular weight cutoff at 30 kDa. The ultrafiltration was repeated four times to obtain purified NEP_1-40_-MP-NPs.

Alexa-HSA was conjugated as described previously [39]. Briefly, 1 mg of the succinimidyl ester fluorophore Alexa Fluor^®^ 555 and 2 mL of HSA (20%) were dissolved in bicarbonate buffer (0.05 M, pH 8.3) and stirred in the dark for 2 h at room temperature. The solution was then purified by dialysis in ultrapure water for 48 h to obtain Alexa-HSA. Alexa-labeled MP-NPs or NEP_1-40_-MP-NPs (Alexa-MP-NPs, Alexa-NEP_1-40_-MP-NPs) were obtained by adding a small amount (5%) of Alexa-HSA to the components at the beginning of preparing MP-NPs or NEP_1-40_-MP-NPs, respectively.

### Characterization of NPs

The average particle size and zeta potential of blank NPs, MP-NPs, and NEP_1-40_-MP-NPs were measured in ultrapure water at 25 °C using dynamic light scattering (Malvern Zetasizer Nano ZS, Malvern, UK). NPs were stained with uranyl acetate and examined using transmission electron microscopy (JEM-1200EX, Japan).

Fourier-transform infrared spectroscopy (IRAffinity-1S, Shimadzu, Japan) was used to analyze MP-PEG and NEP_1-40_-MP-NPs. Samples were blended with KBr at a ratio of 1:100 (w/w). KBr discs were produced under hydraulic pressure of 10,000 psi. Spectra were recorded from KBr pellets at a resolution of 4 mm/s over the wavenumber range 500–4000 cm^−1^. Experiments were performed three times for each sample. MP, PEG, and a physical mixture of MP and PEG were analyzed in parallel as controls for analysis of the MP-PEG complex. MP, blank NPs, NHS-PEG5000-MAL, NEP_1-40_, and the physical mixture of these four components were analyzed in parallel as controls for analysis of NEP_1-40_-MP-NPs.

The conjugation rate during formation of NEP_1-40_-MP-NPs was analyzed based on fluorescently labeled NEP_1-40_. Ultrafiltration was used to remove un-reacted NEP_1-40_, so the fluorescence of NEP_1-40_ was measured in the filtrate and retained sample in triplicate at an excitation wavelength of 488 nm and emission wavelength of 517 nm using an F-7000 fluorescence spectrophotometer (Hitachi, Tokyo, Japan). It was carried out in in triplicate. The conjugation rate was calculated by determining the total fluorescence of the filtrates (W_free_) and subtracting it from the total fluorescence before ultrafiltrations (W_total_): conjugation rate (%) = (W_total_ − W_free_)/W_total_ × 100%.

To complement this fluorescence approach, we also determined the conjugation rate using HPLC. Measurements were run at 25 °C on a ZORBAX Eclipse XDB-C18 column (5 μm, 250 × 4.6 mm) with gradient elution in a mobile phase of acetonitrile (A) and water (B) in 0.05% trifluoroacetic acid at a flow rate of 1.0 mL/min. The linear gradient (A:B) ran from 5:95 at 0 min to 95:5 at 20 min. The detection wavelength was 214 nm. The conjugation rate (%) was calculated as (W_total_ − W_free_)/W_total_ × 100%, where W_total_ refers to the NEP_1-40_ content in the initial solution and W_free_ refers to the NEP_1-40_ content in the collected filtrate.

### MP encapsulation and release in vitro

Encapsulation efficiency and drug loading rate of MP-NPs were estimated by ultrafiltration. A predetermined volume of MP-NPs was added to an ultrafiltration tube with a 30-kDa molecular weight cut-off, and free MP in the filtrate (W_free_) was determined by HPLC (Agilent 1260 Infinity, USA) with a photodiode array detector. Measurements were performed at 25 °C on a C18 column (5 μm, 250 × 4.6 mm, Kromasil). The mobile phase consisted of acetonitrile and ultrapure water (32:68, v/v) and the detector was set at a wavelength of 254 nm.

The same amount of NP suspension was added to cold acetonitrile and vortexed for 5 min to extract MP. The extract was concentrated by centrifugation at 9500*g* for 5 min at 25 °C. The supernatant was assayed by HPLC to quantify the total MP in the nanoparticle system (W_total_). The total weight of the same amount of dried nanoparticles (W_NPs_) was also measured. The encapsulation efficiency and drug-loading rate were calculated using the following formulas:

$${\text{Encapsulation efficiency }}\left( \% \right)\, = \,{{\left( {{\text{W}}_{\text{total}} - {\text{W}}_{\text{free}} } \right)} \mathord{\left/ {\vphantom {{\left( {{\text{W}}_{\text{total}} - {\text{W}}_{\text{free}} } \right)} {{\text{W}}_{\text{total}} }}} \right. \kern-0pt} {{\text{W}}_{\text{total}} }}\, \times \, 100\%$$

$${\text{Drug loading rate }}\left( \% \right)\, = \,{{\left( {{\text{W}}_{\text{total}} - {\text{W}}_{\text{free}} } \right)} \mathord{\left/ {\vphantom {{\left( {{\text{W}}_{\text{total}} - {\text{W}}_{\text{free}} } \right)} {{\text{W}}_{\text{NPs}} }}} \right. \kern-0pt} {{\text{W}}_{\text{NPs}} }}\, \times \, 100\% .$$

Drug release in vitro was analyzed in phosphate-buffered saline (PBS, pH 7.4) containing 0.3% Tween-80 [40]. Three batches of freshly prepared NEP_1-40_-MP-NPs and free MP were put into separate dialysis bags (mol. wt cut-off, 3500 Da) and suspended in 30 mL of release medium. The sealed vials were put in a water bath at 37 °C with slow stirring. At 0.5, 1, 2, 4, 6, 8, 12, 24, 36, 48, 72, 96, and 120 h, sample (1.0 mL) was removed and replaced with the same amount of fresh medium. The sample was fixed with 2 mL methanol and filtered through a 0.22-μm membrane (Millipore). MP content in all samples was determined by HPLC as described above.

### Cellular uptake of NEP_1-40_-MP-NPs

In order to track intracellular transport of nanoparticles, we prepared Alexa-NEP_1-40_-MP-NPs and, as a negative control, Alexa-MP-NPs. Alexa-NEP_1-40_-MP-NPs emit fluorescence in the red and green channels [40], while Alexa-MP-NPs emit only in the red channel due to the lack of FITC-labeled NEP_1-40_. Experiments were conducted in Nogo-positive RSC-96 cells, with Nogo-negative HUVECs as controls. RSC-96 cells and HUVECs were plated at 5 × 10^4^ cells/well on sterile coverslips in 24-well plates 1 day before the cellular uptake assay. Cultures at ~ 70–80% confluence were washed three times with PBS and then incubated with 1 mL of the same concentrations of Alexa-NEP_1-40_-MP-NPs or Alexa-MP-NPs for 4 h at 37 °C. Cells were rinsed with PBS three times and fixed with paraformaldehyde (4%) for 15 min [41], then stained with 150 μL of DAPI (5 mg/mL) for 8 min. Subsequently, coverslips were sealed with glycerol and observed under a confocal laser scanning microscope, in which the emission of Alexa flour and FITC was set at 580 nm and 519 nm, respectively. Cellular uptake was quantified using NIH Image-Pro Plus 6.0.

### Effects of NEP_1-40_-MP-NPs in a rat model of SCI

A rat model of SCI was established using a modified Allen’s weight drop method [42]. Briefly, Sprague–Dawley rats were anaesthetized with an intraperitoneal injection of chloral hydrate (350 mg/kg). The hair on the back of the rat was removed and the skin was disinfected with iodine at the incision site. A dorsal longitudinal mid-line incision (3 cm) was made over the T9–T11 vertebral region using sterile tools. Fascia and para vertebral muscles were gently dissected to expose the lamina and transverse process. Spinal cord contusion was made by dropping a 15-g rod from a height of 10 cm to directly hit the exposed dorsal side of the spinal cord. The rod was removed immediately after the injury. For the sham group, the T10 lamina was opened and the spinal cord was exposed, but not subjected to spinal cord contusion. The animals were randomly divided into the following nine groups (n = 7/group): (a) SCI (saline), (b) SCI (blank NPs), (c) SCI (MPS), (d) SCI (MP), (e) SCI (MP-NPs), (f) SCI (NEP_1-40_-MP-NPs), (g) Sham (Saline), (h) Sham (MPS) and (i) Sham (MP). Each group received a single intrathecal injection (100 μL) of the corresponding treatment. After intrathecal injection, the incision was closed using 4-0 sutures. Cefazolin (100 mg/kg) was administered by intramuscular injection once a day for 7 days to prevent infection. Rats were kept in warm-water pads until waking up from anesthesia. Manual bladder oppression was performed twice a day until the rats were able to urinate spontaneously.

At 1 day after SCI, serum and spinal cord tissue from each group (n = 3) were collected for the detection of MDA, MPO, and SOD [43–45]. All assays were performed according to the instructions in the ELISA kits. For histological examination, spinal cord tissue samples were collected on days 1, 3, and 7 after SCI and fixed in neutral-buffered formalin (10%), dehydrated through a graded series of ethanol solutions and embedded in paraffin. Paraffin sections (5 μm) were prepared and stained with hematoxylin and eosin.

To assess trabecular bone microstructure in rats treated with NEP_1-40_-MP-NPs, animals were sacrificed on day 14 after SCI. The left femur was carefully separated from the surrounding soft tissue and fixed in formalin at room temperature for 24 h. The skeletal microarchitecture (n = 3) was analyzed using high-resolution micro-computed tomography (SIEMENS Healthcare, Berlin and Munich, Germany). Measurement parameters were voltage, 80 kV; current, 80 μA; exposure time, 2.96 s; total rotation angle, 360°; and rotation increment angle, 0.5; scanning resolution, 14 μm/slice. Femur specimens were placed in a 20-mm-diameter sample tube, perpendicular to the scanning axis with a total reconstructed height of 12 mm. One scan took approximately 30 min. Scans were transferred to a workstation for 3-D reconstruction. A cylindrical volume of interest with 3.0-mm diameter and 3.0-mm height was defined for analysis of bone mineral density (BMD), bone volume normalized to total volume (BV/TV), bone surface normalized to bone volume (BS/BV), trabecular thickness (Tb. Th), trabecular number (Tb. N), and trabecular separation (Tb. Sp).

The Basso–Beattie–Bresnahan locomotor rating scale (0–21 points) was used to evaluate the neuromotor function in all rats in a quiet environment at 1, 3, 7, 10, 14, 21 and 28 days after SCI. Two trained technicians blinded to experimental groups performed the assessments. Animals were exposed to an open field of a square plastic box (100 × 100 × 5 cm) for 4 min. Scores were assigned based on movement coordination of limb, paw placement and tail balance [46]. No obvious movement of the hind legs was counted as 0 points. Animals were scored with 21 points if they showed normal locomotion, continuous walking on the paws with a bent tail, normal motor co-ordination for the fore and hind limbs, trunk stability, floor gripping with the toes while moving forward, and alignment of the paws with body movement.

### Statistical analysis

Results are expressed as mean ± standard deviation (SD) and analyzed using GraphPad Prism 6.0 (GraphPad Software, La Jolla, CA, USA). Statistical comparisons between different groups were conducted by one-way analysis of variance (ANOVA). Differences between groups were considered statistically significant when *P *< 0.05.

## Additional file

**Additional file 1: Figure S1.** Comparison of fluorescence intensity in (a) retained sample and (b) filtrate after sequential ultrafiltrations. Results are mean ± SD (n = 3). **Figure S2.** Hemolytic rates of different formulations. Results are mean ± SD (n = 3).

## Abbreviations

BSA: bovine serum albumin
DMEM: Dulbecco’s Modified Eagle medium
HSA: human serum albumin
MDA: malondialdehyde
MP: methylprednisolone
MP-NPs: methylprednisolone-loaded nanoparticles
MPO: myeloperoxidase
MPS: methylprednisolone sodium succinate
MTT: 3-(4,5-dimethylthiazol-2-yl)-2,5-diphenyltetrazolium bromide
NEP_1-40_-MP-NPs: NEP_1-40_-modified nanoparticles loaded with methylprednisolone
PBS: phosphate-buffered solution
PDI: polydispersity index
SOD: superoxide dismutase
