# Supplementary material for: NEP1-40-modified human serum albumin nanoparticles enhance the therapeutic effect of methylprednisolone against spinal cord injury
Source: J Nanobiotechnology. 2019 Jan 22;17:12. doi: 10.1186/s12951-019-0449-3 (PMC6341626; doi:10.1186/s12951-019-0449-3)
Supplement: Supplementary file 1 — Additional file 1: Figure S1. Comparison of fluorescence intensity in (a) retained sample and (b) filtrate after sequential ultrafiltrations. Results are mean ± SD (n = 3). Figure S2. Hemolytic rates of different formulations. Results are mean ± SD (n = 3). [file 12951_2019_449_MOESM1_ESM.docx]

# Additional files

In vitro toxicity detection by hemolysis assay

The hemolytic potential of NPs was evaluated using red blood cells (RBCs) from Sprague-Dawley rats. RBCs were freshly collected and rapidly stirred with a bamboo stick to prevent clotting. RBCs were centrifuged at 2,000 *g* for 8 min for five times and re-suspended using 10 mL PBS. Then MPS, MP-NPs, NEP_1-40_-MP-NPs or blank-NPs (500 μL) were incubated at concentrations of 0.5, 1.0, 1.5, or 2.0 mg/mL with 500 μL 2% RBC suspension at 37 °C for 2 h. Blood compatibility assays in PBS and in 1% Triton X-100 (v/v) served, respectively, as negative and positive controls. All samples were centrifuged at 2,500 *g* for 5 min. The supernatants were measured for hemoglobin content using spectrophotometric detection at 570 nm. The hemolysis rate (%) was calculated according to the following formula: hemolysis rate (%) = (A*_sample_* - A*_negative control_*)/(A*_positive control_* - A*_negative control_*) ×100%.

Figure S1


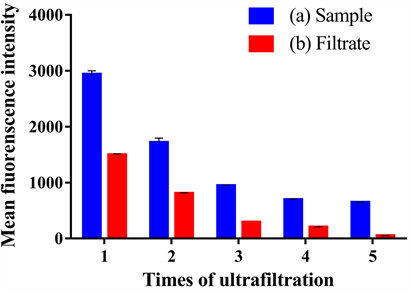


**Figure S1.** Comparison of fluorescence intensity in (a) retained sample and (b) filtrate after sequential ultrafiltrations. Results are mean ± SD (n=3).

Figure S2


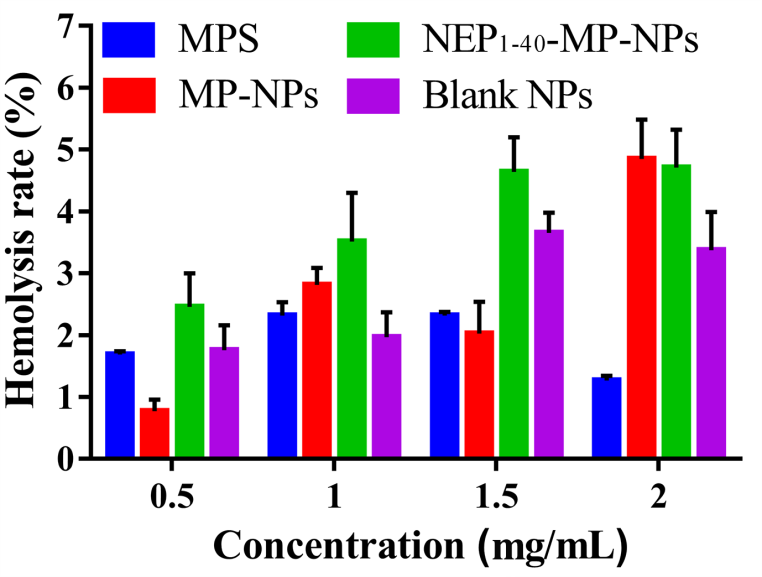


**Figure S2.** Hemolytic rates of different formulations. Results are mean ± SD (n=3).
